# Beyond survival: Practical wellness tips during the 2019 coronavirus disease pandemic

**DOI:** 10.1017/cem.2020.433

**Published:** 2020-06-24

**Authors:** Tetyana Maniuk, Garrick Mok, Nicholas Schouela, Lisa Thurgur, Michael Ho, Lisa Fischer, Shahbaz Syed

**Affiliations:** * Department of Emergency Medicine, University of Ottawa, Ottawa, ON

**Keywords:** COVID-19, emergency medicine, pandemic, physician wellness

Everything changed by one person going to the market. Who could have predicted the domino effect of a daily global occurrence having such devastating consequences? In emergency medicine, we are at the front lines of this pandemic and brave it with one simple mantra: to take care of those in need. However, just like the world around us, things have changed.

Suddenly, the air we breathe sparks uncertainty and fear. Hospital protocols change between bathroom breaks. Gloves and masks, often discarded without a second thought, are causing international political tension. News around the world shows the increasing number of fallen colleagues. Pre-pandemic, the health and wellness of emergency teams was already fragile. Now added to the baseline stress of our profession is the moral injury of ethical dilemmas, grief of isolation, loss of normalcy, and fear of the unknown. Healthcare providers, especially emergency teams, are at high risk of mental health consequences during and post-pandemic.^1^ In fact, Lai et al. found in a survey of 1,257 healthcare workers across 24 hospitals in China taking care of 2019 coronavirus disease (COVID-19) patients that 50% of the respondents had symptoms of depression and anxiety, 72% indicated acute distress, and 34% indicated insomnia.^2^

Thus, we must not forget to take care of ourselves. As Dr. Shem wrote^3^:

“At a cardiac arrest, **the first** procedure is to **take your own pulse**.”

The following are five evidence-based techniques shown to decrease the development of symptoms of depression and anxiety, and how the Department of Emergency Medicine (DEM) in Ottawa has strived to incorporate them.

## MINDFULNESS MEDITATION

*Mindfulness meditation* is a broad term for a variety of meditative practices to assist in grounding individuals in the present; meditation, in general, is hypothesized to modulate the central and peripheral nervous systems, resulting in improved self-regulation and decreased stress.^4^

Mindfulness meditation has shown to be beneficial to brain development and tackling various mental health symptoms. A literature review on nurses and nursing students by van der Riet et al. found that meditation was effective in managing and preventing workplace stress and burnout, while increasing empathy and well-being.^5^ Parsons et al. endorse including mindfulness training into residency programs (Level 1B, Grade B recommendation).^6^

To assist in accessing this resource, our department and university have kept a list of local resources that individual members can access to start, or continue, their own meditation practice.

### Actionable items

Practice mindfulness during stressful situations through box breathing: inhale for 4 seconds, hold for 4 seconds, exhale for 4 seconds, and then hold for 4 seconds. This technique activates the parasympathetic nervous system and decreases cortisol levels.^4^ Utilize “guided meditation,” which is easy to find on any streaming platform; two popular and well-regarded phone apps that offer introductory programs are “Headspace”^7^ and “Calm.”^8^

## EXERCISE

The current Canadian Physical Activity guidelines recommend 150 minutes of moderate to vigorous physical activity per week in increments of 10 minutes or more for ages 18 years and over.^9^ Studies show that exercise has similar modulating effects as antidepressants by increasing sensitivity to norepinephrine, serotonin, and neurotrophic factors, regulating HPA-axis activity, and decreasing inflammatory markers.^10^ In fact, a study of 7,197 surgeons following the recommended exercise guidelines led to decreased scores on burnout and increased professional quality of life measures compared with controls.^11^

Group exercise sessions can be considered to help achieve this goal. For example, our DEM group has organized peer-led virtual yoga and bodyweight exercise sessions for all staff, residents, nurses, and administrators. These workouts included minimal equipment and provided exercise-modifications to include all levels of fitness. The workouts were led by members of our department who are passionate about fitness. These sessions had an anecdotal increase in group morale while promoting healthy habits and virtual socialization.

### Actionable items

Ensure 150 minutes of exercise per week, which can be done in 10-minute increments; there are many fitness programs available online and apps on cell phones to guide you. For example, a popular free phone app accessible on both Android and iOS is “Nike Training Club.”^12^ Departments can also organize virtual exercise sessions led by peers who are passionate about fitness.

## LIMIT SUPERFICIAL SOCIAL MEDIA, DEEPEN DIGITAL SOCIAL CONNECTIONS

With continuous updates and breaking news, it is easy to fall into the trap of endless scrolling on social media. Unfortunately, superficial use of social media has been associated with deleterious effects on mental health, including increased depression and anxiety.^13^ While it is important to limit social media, it is also imperative to find ways to deepen connections with your loved ones. One systematic review of prisoners and their parents found that use of digital communication (e.g., calling, email) maintained strength of emotional attachment over long distance.^14^ In our departmental group, we created a virtual “program director social,” where residents took part in virtual activities (e.g., karaoke, trivia, multiplayer games) with our program directors.

Another option is creating a “Buddy System,” which tasks a buddy to do daily check-ins, ask whether basic needs (e.g., exercise, eating, sleeping) are being met, and do inquiries about mental health. Greenberg et al. included social check-ins and support as part of their recommendations during the COVID-19 pandemic.^15^

Many centres use mentorship or coaching teams, including our own department. In addition to established mentor groups, we also implemented a vertical resident mentorship group (i.e., creation of small resident groups consisting of all resident years). The goal of vertical mentorship is to provide peer support across the years of residency.

### Actionable items

Set a limit on social media: current research suggests more than 30 minutes of social media per day is detrimental to mental health.^13^ Instead, video chat or call a loved one. Establish a buddy system to check in with a loved one.

## DIET

Like exercise, a healthy diet affects almost all aspects of health, including mental.^16^ Although the causal relationship is unclear between diet and mental health, studies support that certain diets, such as the Mediterranean diet, can reduce depressive and anxiety symptoms.^16^ For those new to cooking, now may be a good time to learn. One strategy used in our department was the creation of a cookbook through the collection of recipes submitted by residents. This cookbook provides easy-to-follow recipes that are healthy for the body and mind.

### Actionable items

Experiment with new recipes and share your favourites among friends. A good place to start are recipes that follow the Mediterranean diet. One of the bases of this diet is to limit highly processed foods and red meat.^16^ For example, quinoa and salmon with a side of vegetables would be a delicious meal that follows the Mediterranean diet.

## THERAPY AND COUNSELLING

Finally, today's healthcare providers are experiencing an unprecedented stress. There is grief surrounding the loss of normalcy, moral injury of ethical dilemmas, and the wear and tear of increased workload.^15^ McMahon et al. analysed 54 interviews of 34 providers during the peak of the Ebola outbreak in Sierra Leone and 1 month after; these interviews revealed feelings of loneliness, loss of social connect, and mistrust within the healthcare system.^17^ One recommendation from this study is to have timely access to psychosocial support for healthcare providers during future public health emergencies.

Already, studies are showing negative mental health outcomes to healthcare providers during the COVID-19 pandemic.^2^ In fact, a task force focused on supporting emotional well-being in New York City's healthcare providers made access to psychosocial and mental health support a top priority.^18^ Residents in our department have access to counselling services locally through the university and an outpouring of support from various mental health providers nationally who are offering frontline workers psychosocial support free of charge.

### Actionable items

**Figure 1.**
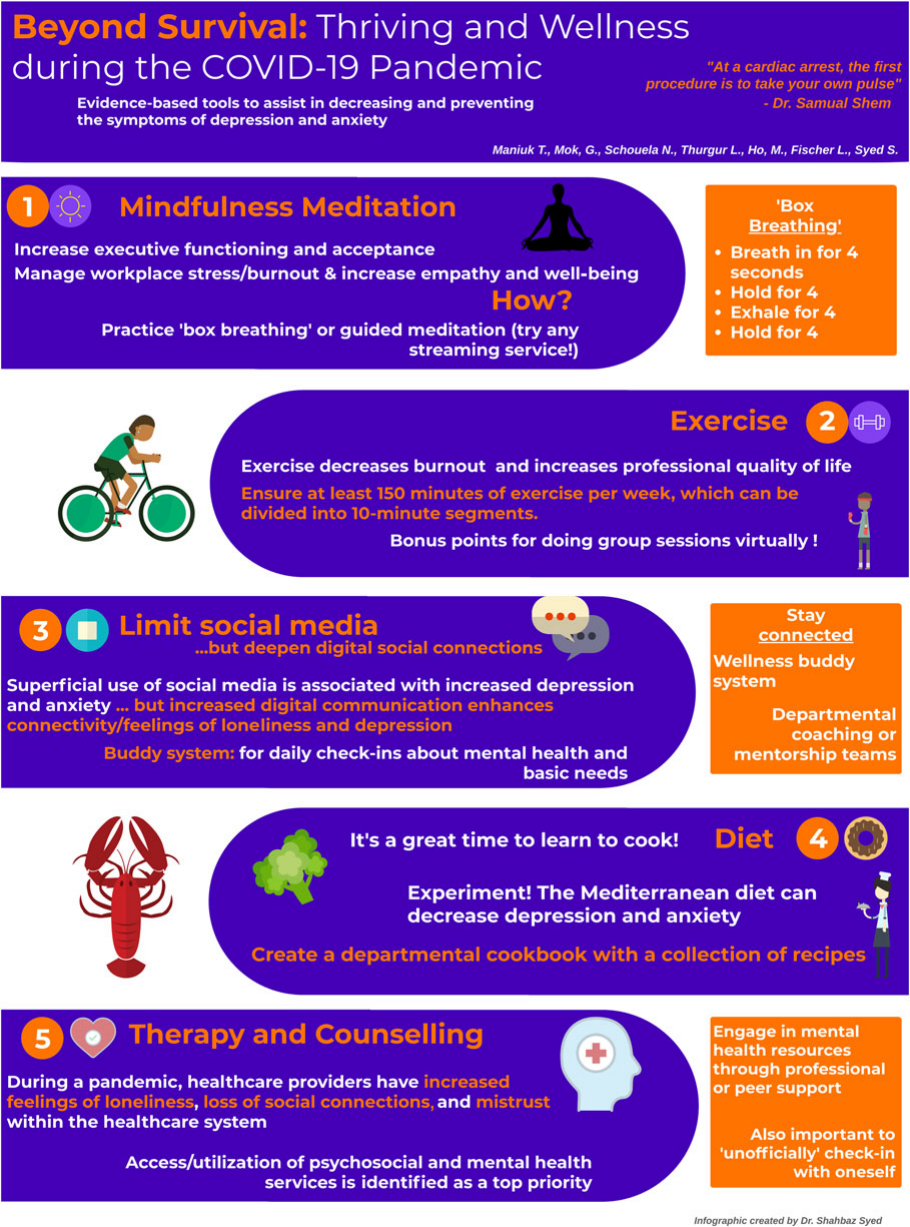
Evidence-based tools to help cope with depression and anxiety.

Engage in mental health resources through peer support or through a mental health professional. While there are numerous supports available officially, it is also important to check in unofficially with oneself. One way that the military has aided this is by creating the “stress continuum,” which divides reactions to stressful situations into colour zones and outlines specific treatments for each zone (Figure 1).

In conclusion, it is apparent, from past experiences and incoming data from the current pandemic, that the mental health of many healthcare providers will be affected. The consequences of an unwell health force may have a significant impact on the Canadian public. It is critical to develop good coping strategies at both the personal and organizational levels. This article highlights some of the ways that our DEM group has accomplished this.

## Supplementary material

For supplementary material accompanying this paper visit http://dx.doi.org/10.1017/cem.2020.433. click here to view supplementary material
