# Supplementary material for: Beyond survival: Practical wellness tips during the 2019 coronavirus disease pandemic
Source: CJEM. 2020 Jun 24:1–5. doi: 10.1017/cem.2020.433 (PMC7369339; doi:10.1017/cem.2020.433)
Supplement: Supplementary file 1 [file S1481803520004339sup001.docx]

**Beyond survival: Practical wellness tips during the COVID-19 pandemic**

**Appendices**

# Appendix A: References

11. Shanafelt T, Oreskovich M, Dyrbye L, Satele D, Hanks J, Sloan J, et al. Avoiding Burnout: The Personal Health Habits and Wellness Practices of US Surgeons. Annals of Surgery. 2012 Apr;255(4):625–33.

12. Nike Training Club App. Home Workouts & More. [Internet]. Nike.com. [cited 2020 Jun 16]. Available from: https://www.nike.com/ca/ntc-app

13. Hunt MG, Marx R, Lipson C, Young J. No More FOMO: Limiting Social Media Decreases Loneliness and Depression. Journal of Social and Clinical Psychology. 2018 Nov 8;37(10):751–68.

14. Cagle JG, Munn JC. Long Distance Caregiving: A Systematic Review of the Literature. J Gerontol Soc Work. 2012;55(8):682–707.

15. Greenberg N, Docherty M, Gnanapragasam S, Wessely S. Managing mental health challenges faced by healthcare workers during covid-19 pandemic. BMJ [Internet]. 2020 Mar 26 [cited 2020 Apr 22];368. Available from: http://www.bmj.com/content/368/bmj.m1211

16. Kiecolt-Glaser JK, Jaremka LM, Hughes S. Psychiatry and social nutritional neuroscience. World Psychiatry. 2014 Jun;13(2):151–2.

17. Adan RAH, van der Beek EM, Buitelaar JK, Cryan JF, Hebebrand J, Higgs S, et al. Nutritional psychiatry: Towards improving mental health by what you eat. European Neuropsychopharmacology. 2019 Dec 1;29(12):1321–32.

18. McMahon SA, Ho LS, Brown H, Miller L, Ansumana R, Kennedy CE. Healthcare providers on the frontlines: a qualitative investigation of the social and emotional impact of delivering health services during Sierra Leone’s Ebola epidemic. Health Policy Plan. 2016 Nov;31(9):1232–9.

19. Ripp J, Peccoralo L, Charney D. Attending to the Emotional Well-Being of the Health Care Workforce in a New York City Health System During the COVID-19 Pandemic. Academic Medicine [Internet]. 2020 Apr 17 [cited 2020 Apr 22];Publish Ahead of Print. Available from: http://journals.lww.com/academicmedicine/Abstract/9000/Attending_to_the_Emotional_Well_Being_of_the.97217.aspx

20. Stress Continuum and Decision Matrix [Internet]. [cited 2020 Jun 17]. Available from: <https://www.marforres.marines.mil/General-Special-Staff/COSC/Stress-Continuum-and-Decision-Matrix/>

# Appendix B: Stress Continuum Model


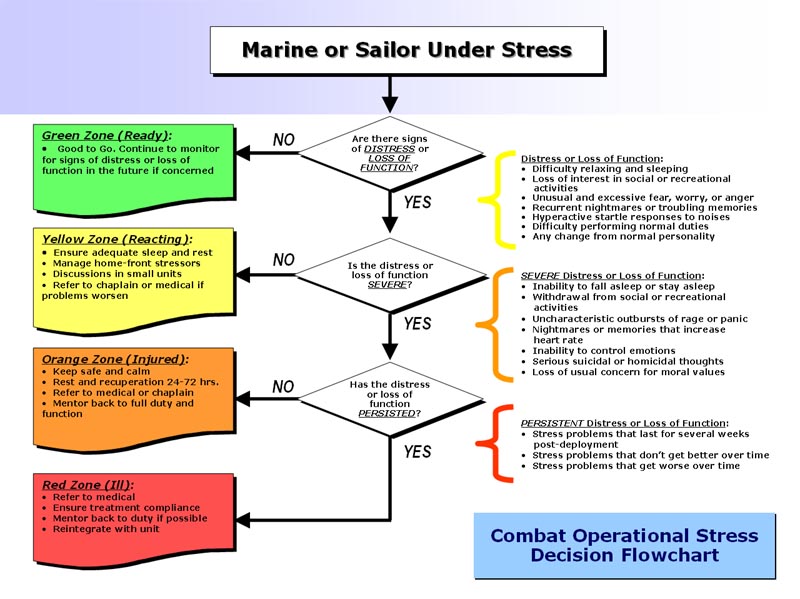


Figure 1. Stress Continuum model (Figure copied from the “Official Website of Marine Corps Forces Reserve” (20)
